# Erosion-free rheumatoid arthritis: clinical and conceptional implications—a BARFOT study

**DOI:** 10.1186/s41927-022-00317-4

**Published:** 2022-12-30

**Authors:** Björn Svensson, Maria L. E. Andersson, Inger Gjertsson, Ingiäld Hafström, Sofia Ajeganova, Kristina Forslind

**Affiliations:** 1 Department of Clinical Sciences Lund, Rheumatology, Faculty of Medicine, Lund University, Lund, Sweden; 2 Spenshult Research and Development Center, Halmstad, Sweden; 3 Department of Rheumatology and Inflammation Research, Institute of Medicine, Sahlgrenska Academy, Gothenburg University, Gothenburg, Sweden; 4 Division of Gastroenterology and Rheumatology, Department of Medicine Huddinge, Karolinska Institutet, Karolinska University Hospital, Stockholm, Sweden; 5 Department of Clinical Sciences, Rheumatology Division, Universitair Ziekenhuis Brussel, Vrije Universiteit Brussel, Brussels, Belgium

**Keywords:** Erosion-free, Rheumatoid arthritis, Radiography, Erosions, ACR criteria, Disease progress, Prognosis

## Abstract

**Background:**

Bone erosions may appear early or later during rheumatoid arthritis (RA), causing joint damage and functional impairment. However, in some patients erosions do not occur, even after several years of disease. This study evaluates the prevalence, clinical relevance and possible predictors of erosion-free RA.

**Methods:**

Six hundred and eight patients from an early RA cohort (BARFOT) having radiographs of hands and feet at inclusion and after 1, 2, 5 and 8 years were studied. Clinical and functional assessments were performed on all these time-points.

**Results:**

In all, 144 patients (24%) did not develop erosions up to 8 years follow-up (Never erosive group), while 464 patients (76%) had erosions on one or more assessments (Ever erosive group). At diagnosis, the patients in the Never erosive group were significantly younger, satisfied fewer ACR criteria, and were less frequently RF- and/or anti-CCP- positive compared with those in the Ever erosive group. The Never erosive patients had consistently more tender joints, lower erythrocyte sedimentation rate (ESR) and, from two years and onwards, fewer swollen joints. Absence of rheumatoid factor (RF) and/or anti-CCP were strong independent predictors for erosion-free disease. The erosion-free patients were less frequently treated with DMARDs and/or prednisolone.

**Conclusions:**

One-quarter of the patients was erosion-free during eight years in this early RA cohort. Erosion-free patients had a less severe disease course as to disease activity and were more often seronegative compared with those with erosive disease. The results suggest that non-erosive RA represents a milder form of RA.

## Background

Rheumatoid arthritis (RA) is a systemic autoimmune disease of unknown aetiology, characterized by inflammation in joints and other organs [1]. In most patients, bone erosions resulting in joint destruction appear during the course of disease.

Early therapy has been shown to be effective in achieving control of disease activity and retarding radiographic damage [2]. A proportion of patients never develops erosions and identifying these patients at an early stage may be of relevance for the choice of treatment.

Conventional radiography of the hands and feet is the recommended imaging method used for diagnosis and prediction of joint damage in RA [3]. The first erosions may appear after varying periods of time from symptom onset. Previous studies suggest that erosions in most cases appear within two years of diagnosis [4].

In a recent study on the distribution of erosions in hands and feet in RA we noticed that a number of patients did not seem to develop any erosion [5]. However, that this may occur has already been reported in the latter part of the twentieth century. Thus, Mottonen et al. found that, after two years, 24% of 58 patients were free from erosions [6]. Nevertheless, the prevalence of erosion-free RA is not fully known. In a prospective observational study of 271 patients with established RA followed for over 2 years, Liao et al. [7] found that 21% remained erosion free. In a systematic literature review, Amya-Amya et al. reported the prevalence of non-erosive RA ranged 11–85% in the studies of varying design and follow-up time [8].

Against this background it seemed to be of interest to study the prevalence, clinical relevance and possible predictors of erosion-free RA in a long-term cohort study of patients with early RA.

## Methods

### Patients

In total, 2857 patients were included in the BARFOT (Better Anti-Rheumatic FarmacOTherapy) early RA cohort between the years 1992 and 2006 [9]. All patients should, according to the protocol, fulfil the American College of Rheumatology (ACR) 1987 revised criteria for the classification of RA [10] and should have a disease duration of no more than 12 months.

Radiographs of hands and feet were performed at inclusion on 1155 patients from five of the six BARFOT centres, 592 were included in the 1990s and 563 in the 2000s. Six hundred and eight of the 1155 patients had radiographs performed at baseline and all pre-determined follow-up visits after 1, 2, 5 and 8 years and constitute the study material.

Of the 547 patients not included in the study due to incomplete radiographs, 124 had died. Among the remaining 423 patients, 84 had left the study from a variety of defined reasons and 25 from unknown reasons.

### Clinical assessments

Disease activity was assessed by the composite index Disease Activity Score, calculated on 28 joints (DAS28; range 0–9.4, remission defined as DAS28 < 2.6) [11]. DAS28 includes the erythrocyte sedimentation rate (ESR; 0–150 mm/h), the number of swollen joints (SJC; range 0–28), the number of tender joints (TJC; range; 0–28) and the patient’s global assessment of general health (PatGA) measured on a visual analogue scale (VAS; range 0–100 mm). Pain was assessed by a similar VAS (0–100 mm).

Daily life function was measured by the Swedish version of the Stanford disability index Health Assessment Questionnaire (HAQ), (range 0–3) [12].

Rheumatoid factor (RF) was measured according to the local laboratory standards. Antibodies to cyclic citrullinated peptides (anti-CCP) were analyzed either on baseline serum from the biobank or directly at inclusion and detected using the ELISA CCP2 test (Euro-Diagnostica, Malmö, Sweden) and a positive test was defined according to the various laboratory standards. Information on the presence of RF was available in 602 and of anti-CCP in 426 patients.

### Radiographic assessment

Posterior-anterior radiographs of the hands and feet were assessed according to the van der Heijde modification of the Sharp score (SHS) where 32 joints in the hands and 12 in the feet are assessed calculating total SHS (range 0–448), erosion score (ES) (range 0–280), and joint space narrowing score (JSN) (range 0–168) [13].

The radiographs were read by one of two experienced readers. Double readings of a fraction of films showed good agreement between the two readers. The intra-class correlation coefficient for SHS was excellent (0.940–0.998). Furthermore, the agreement between two observers in identifying the presence or absence of erosions was also calculated. Kappa proved to be 0.80 implying substantial agreement.

Erosive disease was defined as presence of erosions (an erosion score ≥ 1) on radiographs of the hands (hands and wrists) and/or feet at baseline or any follow-up visit. Erosion-free RA was defined as absence of erosions at all time-points.

Early onset of erosions was defined as the appearance of erosions at baseline or at one year and late onset as appearance of erosions at 2, 5 or 8 years.

### Medical treatment

In the 1990s, initial disease modifying anti-rheumatic drug (DMARD) monotherapy and early use of low-dose glucocorticoids was recommended in Sweden. In 1999, biological treatment was introduced and consequently, in the beginning of the 2000s, treatment options increased, and therapeutical strategies changed.

In some patients, treatment start was not recorded until the three months visit even if started soon after inclusion. This means that treatment start refers to therapy prescribed at inclusion even if not recorded until the first visit thereafter, the 3 months visit.

### Statistics

Statistical analyses were performed using the SPSS version 21.0 statistical software (IBM Corp., Armonk, NY, USA). To test the differences between groups the Student’s t-test or the Mann–Whitney U test was used for continuous variables, and the chi-squared test was used for proportions. For tables larger than 2 × 2, standardized residuals were computed to evaluate differences between expected and observed values. If the standardized residual is ≥  + 2 or ≤ − 2, the difference is considered significant. Spearman’s correlation coefficient was used to assess relationships between two continuous variables.

Multiple logistic regression analyses were performed to examine possible associations between baseline variables and erosion-free disease. Age, gender, disease duration, number of satisfied ACR-criteria, smoking habits, RF, anti-CCP, DAS28, number of swollen and tender joints, PatGA, ESR, VAS pain, HAQ as well as initial treatment with DMARDs and/or prednisolone were considered and tested in bivariate analyses. Collinearity was considered if two variables correlated ≥ 0.8. Variables associated with erosion-free disease with a bivariate significance level of *p* < 0.25 were introduced into the multiple logistic regression models [14]. Otherwise, the significance tests were two tailed and conducted at the 0.05 level of significance.

## Results

The 608 study patients did not statistically differ in baseline characteristics or in radiological scores from the 423 patients (excluding those who had died) with incomplete radiographic data (data not shown).

Similar proportions of patients with and without complete radiographic data were included in the 1990s and 2000s *p* = 0.15.

The 608 patients were divided into two groups, the Never erosive group consisting of 144 patients (24%) who did not show erosions at baseline or at any of the pre-determined follow-up visits at 1, 2, 5 and 8 years, and the Ever erosive group consisting of 464 patients (76%) with erosions consistently or at some point(s) in time.

The patients in the Never erosive group fulfilled fewer ACR1987 criteria than those in the Ever erosive group, less than four criteria in 5 versus 1%, four in 49 versus 29%, five in 39 versus 52%, six in 7 versus 16% and seven in 0 versus 2%, respectively, *p* = 0.001.

Sixty-four percent of the Never erosive patients were recruited in the 1990s and 36% in the 2000s (*p* = 0.04) while 50% of the Ever erosive patients were included in each millennium.

Nine patients satisfied less than four of the seven ACR criteria for the diagnosis of RA, six belonging to the never erosive group and three to the erosive group, and thus represent deviations from the study protocol. Seven of these nine were anti-CCP- negative, six were women and two of eight had large joint onset. These patients were judged as having RA based on the clinical picture at onset and onwards as well as absence of symptoms or signs of any alternative rheumatic diagnosis.

The frequency of large joint onset was similar in the two groups, 35 patients (27%) in the Never and 125 (31%) in the Ever erosive group, *p* = 0.46.

### Baseline differences

Table 1 shows that the patients in the Never erosive group were, compared with those in the Ever erosive group, significantly younger, less frequently RF or anti-CCP positive, had lower ESR and lower joint space narrowing (JSN) score.

**Table 1:** Demographic and clinical differences at baseline between Never and Ever erosive patients

	Never erosive		Ever erosive		Diff
	n = 144		n = 464		
	Mean (SD)	n (%)	Mean (SD)	n (%)	*p*-value
Inclusion age	52 (15)		55 (13)		0.006
Female gender		94 (65)		320 (69)	0.41
Dis dur, months	6 (3)		6 (3)		0.15
RF		64 (44)		316 (69)	0.001
Anti- CCP		36 (31)		217 (70)	0.001
Current smoker		31 (22)		126 (27)	0.20
DAS28	4.9 (1.4)		5.1 (1.2)		0.16
SJC28	9 (6)		10 (6)		0.08
TJC28	8 (7)		7 (6)		0.050
PatGA	45 (27)		45 (25)		0.97
Pain	45 (25)		47 (25)		0.34
ESR	26 (21)		34 (24)		0.001
HAQ	0.91 (0.66)		0.90 (0.59)		0.91
SHS	0.40 (1.51)		7.19 10.52)		0.001
Erosion score	0		2.52 (4.07)		na
JSN	0.44 (1.58)		4.68 (7.79)		0.001

Dis dur: Disease duration, RF: Rheumatoid Factor, Anti- CCP: anti-cyclic citrullinated protein, DAS28: 28-joints Disease Activity Score, SJC: swollen joint count, TJC: tender joint count, PatGA: patient´s global assessment, ESR: erythrocyte sedimentation rate, HAQ: Health Assessment Questionnaire, SHS: Sharp van der Heijde score, JSN: joint space narrowing score, na: not applicable

### Disease course

**Fig. 1:**
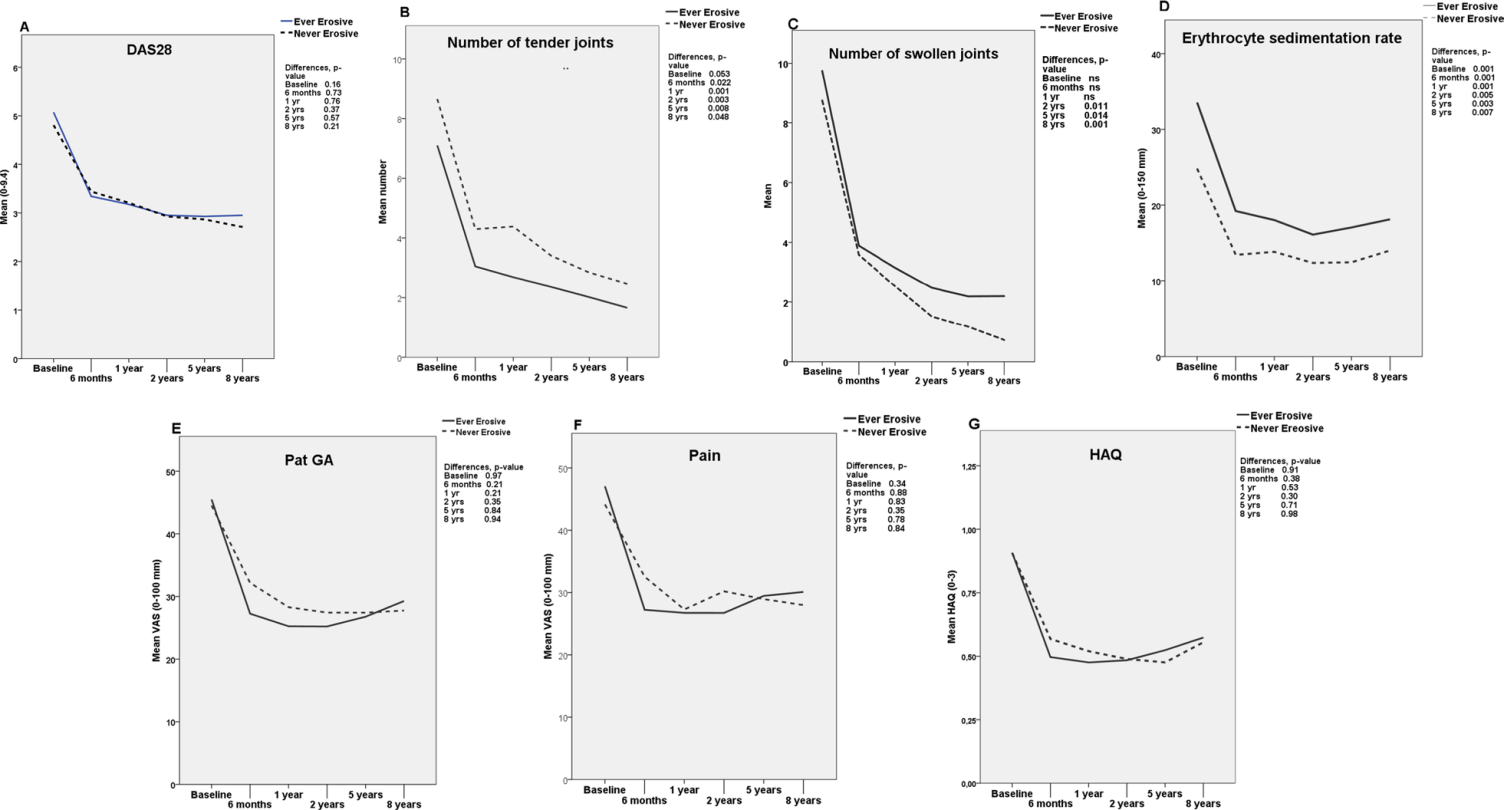
The graphs show the disease course in the Never and Ever erosive groups from baseline to eight years regarding 28-joints Disease Activity Score (DAS28) (**A**), number of tender joints (**B**), number of swollen joints (**C**), erythrocyte sedimentation rate (**D**), visual analogue scale (VAS) patient global assessment (PatGA) (**E**), VAS pain (**F**) and Health assessment questionnaire (HAQ) (**G**). On the Y-axis we present the mean values of the measured data

The disease course is shown in Fig. 1A–G.

The course from six months and onwards was similar in the Never and Ever erosion groups regarding DAS28, PatGA, VAS pain and HAQ. However, the Never erosive group had, compared with the Ever erosive, consistently lower ESR, more tender joints and, from two years and onwards, a lower number of swollen joints.

At the end of the eight-year study, the patients in the Never erosive group still had significantly lower ESR, more tender joints and also fewer swollen joints than the patients in the Ever erosive group.

Twenty-eight percent of the patients in the Never erosive group were in DAS28 remission at all follow-up visits (sustained remission) vs. 15% in the Ever erosive group, *p* = 0.003.

The Never erosive group had a significantly lower JSN score than the Ever erosive group at baseline, 1, 2, 5 and 8 years, mean 0.4 versus 4.7, 0.7 versus 8.0, 1.0 versus 10.9, 1.8 versus 16.4 and 3.0 versus 21.3, respectively, *p* = 0.001 for all comparisons. At the same time-points, the mean erosion score for the Ever erosion group was 2.5, 4.6, 6.6, 9.2 and 10.0.

### Treatment

Crosstabulations of the use of conventional DMARDs (cDMARDs), biologic DMARDs (bDMARDs) and prednisolone (glucocorticoid) are shown in Table 2. The main differences between the groups were that, at all-time points, a significantly higher proportion of patients in the Never erosion group did not receive any treatment with DMARDs or prednisolone.

**Table 2:** Crosstabulations of DMARD and Prednisolone treatment in the Never and Ever erosion groups over 8 years

	Never erosions		Ever erosions		Diff
	n = 144		n = 464		
	N (%)	SR*	N (%)	SR*	*p*- value
*Treatment start*					
No DMARD No Pred	22 (15)	≥ + 2	33 (7)		0.003
cDMARDs	104 (72)		394 (85)		
bDMARDs	6 (4)		8 (2)		
Pred only	12 (8)		29 (6)		
*Treatment 0.5 yr*					
No DMARD No Pred	21 (16)	≥ + 2	37 (9)		0.003
cDMARDs	93 (70)		365 (84)		
bDMARDs	8 (6)	≥ + 2	9 (2)		
Pred only	10 (8)		22 (5)		
*Treatment 1 yr*					
No DMARD No Pred	30 (21)	≥ + 2	40 (9)		0.001
cDMARDs	96 (68)		379 (84)		
bDMARDs	9 (6)		20 (4)		
Pred only	7 (5)		11 (2)		
*Treatment 2 yrs*					
No DMARD No Pred	35 (26)	≥ + 2	45 (10)	≤ − 2	0.001
cDMARDs	80 (58)	≤ − 2	349 (78)		
bDMARDs	10 (7)		36 (8)		
Pred only	12 (9)	≥ + 2	16 (4)		
*Treatment 5 yrs*					
No DMARD No Pred	54 (39)	≥ + 2	68 (15)	≤ − 2	0.001
cDMARDs	61 (44)	≤ − 2	303 (68)		
bDMARDs	14 (10)		61 (14)		
Pred only	9 (7)		14 (3)		
*Treatment 8 yrs*					
No DMARD No Pred	50 (35)	≥ +2	81 (18)		0.001
cDMARDs	74 (52)		299 (65)		
bDMARDs	7 (5)	≤ − 2	58 (13)		
Pred only	12 (8)		19 (4)		

*SR—Standardized residual: A SR ≥ + 2 or ≤ − 2 is judged to be significant

DMARD: disease modifying anti-rheumatic drug, Pred; prednisolone, cDMARD: conventional DMARD, bDMARD: biologic DMARD, yr; year, yrs; years

### Rheumatoid factor and anti-CCP in Never and Ever erosive patients

RF- and anti-CCP- positivity were significantly less frequent in the Never erosive than in the Ever erosive group, 44 versus 69% and 31 versus 70%, respectively, *p* = 0.001 for both comparisons, Table 1.

Never erosive RF- or anti-CCP positive patients did not significantly differ from those negative for these autoantibodies as to demographics or clinical data at baseline and 8 years. The exception was that the mean VAS PatGA was significantly higher at baseline in the anti-CCP positive group, 54 versus 42, *p* = 0.019.

Ever erosive RF- positive patients differed at baseline from those RF- negative in having higher ESR, 35 versus 29 mm, *p* = 0.012, and similarly, anti-CCP- positive patients differed from anti-CCP- negative in also having higher ESR, 37 versus 28 mm, *p* = 0.002. At eight years, RF- positive patients had, compared with those negative, higher mean ESR, 19 versus 16 mm *p* = 0.009 and higher mean number of swollen joints, 2.5 versus 1.5, *p* = 0.001, and anti-CCP- positive patients had higher mean ESR than those negative, 21 versus 16 mm, *p* = 0.005.

### Time to first erosion

**Fig. 2:**
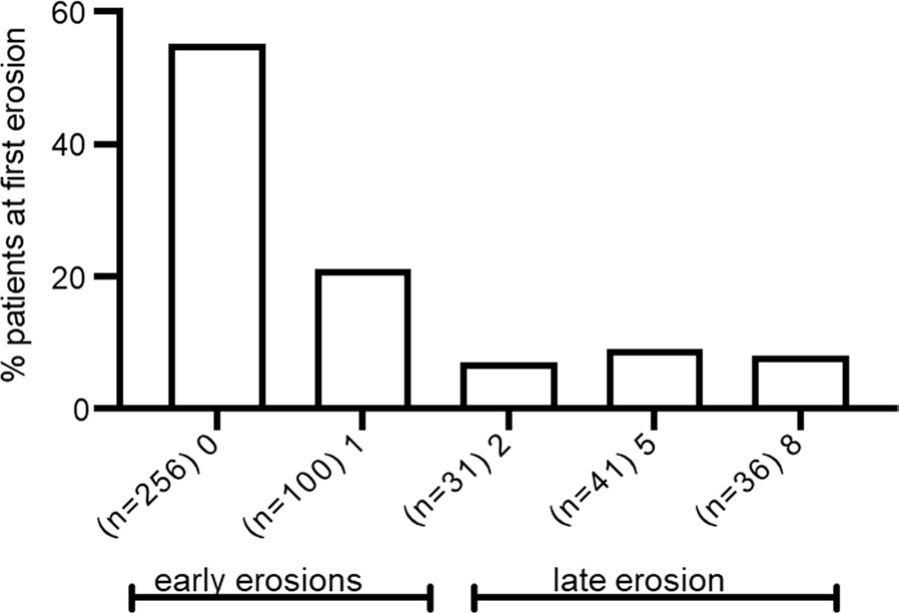
The time to first erosion in the Ever erosive patients (n = 464). Percent of patients with early onset of erosions (at baseline or one year) and with late onset (at two, five or eight years)

Four hundred and sixty-four patients displayed erosions during the eight-year study. Figure 2 displays the time to first erosion, which was similar in the 1990s and 2000s. Early onset of erosions (at baseline and one year) occurred in 77% and late onset (at 2, 5 and 8 years) was seen in 23% of these patients.

The time from symptom onset to diagnosis (disease duration) was mean 6.2 months in the patients included in the 1900s and mean 5.9 in those included in the 2000s, *p* = 0.36.

At baseline, patients with first erosion late in the disease course differed from those with early erosions in being younger, mean 52 versus 56 years, *p* = 0.001, having more tender joints, mean 9 versus 7, *p* = 0.03, higher mean VAS PatGA 50 versus 43 mm, *p* = 0.024 but not mean VAS pain, 50 versus 46 mm, *p* = 0.10, and higher mean HAQ, 1.01 versus 0.87, *p* = 0.034, but lower mean ESR, 29 versus 35, *p* = 0.014. Presence of autoantibodies as well as treatment with DMARDs and Prednisolone were similar in patients with early and late onset of erosions (data not shown).

At eight years, patients with late onset of erosion still had lower mean ESR, 15 versus 19, *p* = 0.008.

### Logistic regression models to assess possible baseline predictors of erosion-free RA

In a multivariate model including rheumatoid factor (RF), absence of RF and a high tender joint count were significant independent predictors of erosion-free disease (Table 3). In a second model substituting RF for anti- CCP, absence of anti-CCP strongly predicted erosion-free disease (Table 4).

**Table 3:** Multiple logistic regression analysis of the associations between baseline variables and erosion-free disease up to 8 years. The model includes rheumatoid factor. 70% of the study patients was included in the model. Nagelkerke pseudo-R^2^ was 0.15

	B	Wald	Sig	Exp(B)	95% C.I.for EXP(B)	
					Lower	Upper
Rheumatoid factor	− .730	5.355	.021	.482	.260	.894
Number of ACR- criteria	− .409	3.630	.057	.664	.436	1.012
Inclusion age	− .007	.648	.421	.993	.976	1.010
Disease duration, months	.052	1.545	.214	1.053	.971	1.143
Current smoking	− .057	.040	.841	.945	.543	1.645
DAS28	− .292	1.963	.161	.747	.497	1.123
SJC28	− .024	.743	.389	.976	.923	1.031
TJC28	.073	5.160	.023	1.076	1.010	1.146
ESR	.003	.143	.705	1.003	.987	1.020
Treatment start	.005	.001	.982	1.005	.640	1.578
Constant	2.267	3.949	.047	9.652		

CI; confidence interval, ACR: American College of Rheumatology, DAS28: 28-joints Disease Activity Score, SJC: swollen joint count, TJC: tender joint count, ESR: erythrocyte sedimentation rate, Treatment start: DMARDs and/or Prednisolone at study start (first visit or 3 months visit)

**Table 4:** Multiple logistic regression analysis of the associations between baseline variables and erosion-free disease up to 8 years. The model includes anti- CCP. 50% of the study patients were included in the model. Nagelkerke pseudo-R^2^ was 0.30

	B	Wald	Sig	Exp(B)	95% C.I.for EXP(B)	
					Lower	Upper
Anti- CCP	− 1.752	25.631	.000	.173	.088	.342
Number of ACR-criteria	−.411	3.335	.068	.663	.427	1.031
Inclusion age	−.015	1.955	.162	.985	.965	1.006
Disease duration, months	.087	2.814	.093	1.091	.985	1.208
Current smoking	.378	1.246	.264	1.459	.751	2.834
DAS28	.040	.021	.884	1.041	.611	1.773
SJC28	−.056	2.589	.108	.946	.884	1.012
TJC28	.039	.947	.331	1.040	.961	1.124
ESR	−.003	.057	.811	.997	.977	1.018
Treatment start	.020	.006	.940	1.021	.601	1.734
Constant	2.072	2.329	.127	7.941		

CI; confidence interval, Anti- CCP: anti-cyclic citrullinated protein, ACR: American College of Rheumatology, DAS28: 28-joints Disease Activity Score, SJC: swollen joint count, TJC: tender joint count, ESR: erythrocyte sedimentation rate, Treatment start: DMARDs and/or Prednisolone at study start (first visit or 3 months visit)

## Discussion

The present study on 608 patients from our early RA cohort [9] showed that 24% were free from erosions at diagnosis and all pre-determined follow-up visits up to eight years. To our knowledge, this study is the first to report the persistent absence of erosions in RA at diagnosis and several pre-defined follow-up visits during an extended disease course. However, erosion-free RA has been addressed previously. Thus, already in 1970 Lawrence summarized some of the studies performed in the 1950th and 1960th in a Heberden Oration [15] and reported that clinical inflammatory polyarthritis was not always erosive. Most of these studies used the ARA (American Rheumatism Association) revised criteria for the diagnosis of RA [16], which as knowledge expanded were shown to include a significant number of patients with diseases other than RA. In 1988 [10], the ACR presented new classification criteria having higher specificity and sensitivity for RA and were used in the present study. These criteria were also used by Liao et al., who reported a similar prevalence of erosion-free disease as that found in our study, 21% of 271 patients followed for two years in a cohort of established RA [7]. In 2015, a systematic literature review of 18 studies, also using the 1987 ACR criteria, reported erosion-free RA of varying frequencies [8]. However, the results of this review are difficult to interpret as in 15 of the 18 studies non-erosiveness was based on radiographs at only one point in time in between inclusion and end of study.

According to the criteria used and the long-term follow-up of these patients, we are convinced that the diagnosis of RA is correct also for the nine patients not fulfilling four or more of the ACR criteria as they remained in the cohort for eight years without a change of diagnosis.

At diagnosis, the erosion-free patients were, compared with the patients with erosions, younger, had less frequently RF or anti-CCP antibodies, had lower ESR, and satisfied fewer ACR criteria. Also, Liao et al. reported, in addition to a short disease duration, lower age and lower frequency of anti-CCP in non-erosive patients [7]. Lower frequencies of autoantibodies were also reported in the study by Amaya-Amaya et al. as well as by others [8].

In the present study, only two baseline variables were associated with erosion-free disease, absence of RF or anti-CCP, and a higher tender joint count. Liao et al., too, found only few baseline predictors, younger age, and shorter disease duration but not absence of RF or anti-CCP, which in several studies has been found to be major predictors of erosive RA [17–19].

As to the tender joint count, previous studies have reported that the tender joint count may not predict radiological progression. Thus, Navarro-Compán et al. reported that, as a single instrument, the tender joint count was not associated with radiographic progression [20]. Similarly, Cheung PP et al. found that the tender joint count was poorly associated with structural progression [21]. In addition, a high tender joint count was reported to be weekly associated with more objective assessments of inflammation, measured by ultrasound [22]. Rather, the tender joint count was primarily associated with pain [22]. In early RA, however, tender joints seemed to be more related to inflammation [23]. In the present study, the non-erosive patients had higher tender joint count and lower ESR throughout the study compared with the erosive patients, which indicates that the tender joint count in our patients were not solely dependent on inflammation.

DAS28 was over the studied period similar in the Never and Ever erosive groups. It is likely that the inflammatory activity despite similar DAS28 was lower in the non-erosive group because the high tender joint count overestimated the disease activity measured by DAS28. This suggestion is supported by the fact that sustained remission over the course of the disease was significantly more frequent among the Never erosive patients.

Increasing evidence indicates an important role for ACPA in osteoclastogenesis resulting in bone erosion formation and bone loss in RA [24–26]. However, also RF has been associated with bone specific effects [24]. It is of major importance to stress that erosive disease may be, at least partly, unrelated to the inflammatory process. To this end, Ten Klooster et al. have shown that even patients with low or moderate disease activity may be at risk of longer-term radiographic damage [27]. Furthermore, in an early RA cohort increased radiological damage was not associated with disease activity over time in anti-CCP positive patients [28].

The mean HAQ disability index in the Never and Ever erosive groups was similar during the entire disease course. This is in accordance with previous observations that “the predominant determinants of HAQ disability in RA are disease activity, pain, and psychosocial factors rather than structural abnormality” [29]. Accordingly, there were no significant differences in mean VAS Pain between the non-erosive and erosive groups.

At all times, the treatment with DMARDs or prednisolone was discontinued in a significantly higher proportion of patients in the Never erosive than in the Ever erosive group. This suggests that the treating rheumatologists found the disease less active in the erosion-free group, which may reflect that non-erosiveness is an expression of a milder form of RA. Similarly, in their cross-sectional study, Amaya-Amaya et al. reported less frequent DMARD therapy in patients with non-erosive arthritis [8].

In the present study, the time to first erosion was one year or less in 77% and two years or more in 23% despite similar symptom duration prior to diagnosis and similar treatment. Some studies report that the majority of patients with RA develop erosions within the first two years [6, 30]. However, in the present study, the first erosions appeared also later up to the eight-year follow-up visit. The patients with late occurrence of erosions had, compared with those with early, a less active disease at inclusion but autoantibodies were present in similar proportion in the late and early erosion groups. After eight years both erosion groups displayed a similar clinical picture except that ESR was significantly higher in the early erosion group. So, it is still unclear whether late appearing erosiveness is a reflection of a milder RA or an effect of early treatment. However, the data suggests that patients with a diagnosis of RA, who are consistently erosion-free for eight years, have a less active disease course than those who, earlier or later, develop erosions. This is consistent with the observation that there is a gradient in disease severity from patients with Rapid Radiographic Progression to patients who never displayed erosions (data not shown). So, with regard to treatment, reliable predictors of non-erosive disease would be of advantage. However, yet such predictors are unavailable in clinical practice. Therefore, treatment of erosion-free RA should still be based on other prognostic factors according to the EULAR recommendations [31].

This study confirms previous observations suggesting that an initially erosion-free RA may remain non-erosive during a long disease course or may become erosive in a later stage. We believe that the demonstrated demographic and clinical differences between Never erosive and Ever erosive patients are not distinct enough to suggest different disease entities but might be explained by different pathophysiological mechanisms for erosions.

### Strengths and limitations

One strength of this study was the long observational period of eight years offering an opportunity to study the course of RA radiographically from disease onset. Another strength was the large sample size and that all the participating patients had radiographs at all pre-defined follow-up visits. A limitation was that the participating patients were enrolled between 1992 and 2006, a period during which medication options and treatment strategies changed. Furthermore, anti-CCP was available in only 70% of the study patients included in the 2000s.

## Conclusions

As many as 24% of patients with incident RA did not develop erosions during a disease course of eight years. These patients were, compared with patients who developed erosions, younger, had a less severe disease course as to disease activity, less frequently autoantibodies, and were less often treated with DMARDs or prednisolone. In view of these observations, erosion-free disease was regarded as a milder form of RA.

## Data Availability

The data sets used are available from the corresponding author on reasonable request.

## Abbreviations

ACPA: Anti-citrullinated protein antibody
ACR: American College of Rheumatology
Anti- CCP: Anti-cyclic citrullinated protein
ARA: American Rheumatism Association
bDMARD: Biologic DMARD
BARFOT: Better Anti-Rheumatic FarmacOTherapy
cDMARD: Conventional DMARD
CI: Confidence interval
DAS28: Disease Activity Score 28-joints
Dis dur: Disease duration
DMARD: Disease modifying anti-rheumatic drug
ES: Erosion score
ESR: Erythrocyte sedimentation rate
EULAR: European League Against Rheumatism
HAQ: Health assessment questionnaire
JSN: Joint space narrowing score
n: Number
na: Not applicable
PatGA: Patient global assessment
Pred: Prednisolone
RA: Rheumatoid Arthritis
RF: Rheumatoid factor
SHS: Sharp van der Heijde score
SJC: Swollen joint count
TJC: Tender joint count
VAS: Visual analogue scale
yr/yrs: Year/years
